# Accuracy of an Overnight Axillary-Temperature Sensor for Ovulation Detection: Validation in 194 Cycles

**DOI:** 10.3390/s25206327

**Published:** 2025-10-13

**Authors:** Yaniv Shpaichler, Alicia Thompson, Benedicte Fromager, Michael Vardi, Rene Ecochard

**Affiliations:** 1 Tempdrop Ltd., Tel Aviv 6706058, Israel; yaniv@tempdrop.com (Y.S.); michael@tempdrop.com (M.V.); 2 Westar OB/Gyn, Mount Carmel Hospital, Columbus, OH 43213, USA; aliciawthompson@gmail.com; 3 CHU de Lyon, 69002 Lyon, France; rene.ecochard@chu-lyon.fr; 4 UMR CNRS 5558, Laboratoire de Biométrie et Biologie Evolutive, Equipe Biostatistique-Santé, Université Claude Bernard Lyon 1, 69100 Villeurbanne, France

**Keywords:** menstrual cycle, temperature, ovulation detection/methods, wearable electronic devices, armband

## Abstract

**Highlights:**

**What are the main findings?**

- An axillary skin temperature sensor (Tempdrop) can accurately determine the timing of ovulation.
- It provides a clear temperature curve.

**What is the implication of the main findings?**

- It is an easy and effective alternative to urine ovulation tests for detecting ovulation.
- It can be used to evaluate the quality of the luteal phase.

**Abstract:**

Several studies have evaluated the reliability of using temperature sensors placed in different locations on the body to identify the day of ovulation. However, such demonstrations are lacking for axillary temperature wearable devices. This study aimed to evaluate the accuracy with which an axillary temperature armband sensor (Tempdrop) identifies the day of ovulation and the fertile window, using the Clearblue Connected Ovulation Test System as the reference method. A total of 194 cycles were analyzed from 125 women that participated in the study between April 2023 and June 2024. The performance parameters were high: the sensitivity (96.8% (95% CI 95.6; 97.7)), specificity (99.1% (98.8; 99.4)), accuracy (98.6% (98.2; 98.9)), positive predictive value (96.8% (95.6; 97.7)) and negative predictive value (99.1% (98.8; 99.4)). Furthermore, the results revealed a remarkably clear and better-than-expected change in temperature around the time of ovulation. This axillary temperature wearable sensor is an effective alternative to urine ovulation tests for determining the timing of ovulation. Another advantage is that it provides a clear temperature curve that can be used to evaluate the quality of the luteal phase.

## 1. Introduction

In recent years, portable electronic devices that analyze physiological changes during the menstrual cycle have emerged as a practical alternative to urine-based ovulation tests for determining the time of ovulation. It is a valuable medical aid for couples who want to accurately identify their fertile period to better plan their sexual intercourse and thus increase their chances of conceiving. Several studies have evaluated the reliability of various sensors placed at different locations on the body [1], namely the ear [2], vagina [3] or upper limb (i.e., wristbands [4,5] and rings [6]) to identify the day of ovulation. However, such demonstrations are lacking for armband devices [7,8].

Portable electronic devices have earned their new status, namely their recognized ability to determine the time of ovulation and fertile window, for several reasons [9]: (1) Wearable devices that measure temperature continuously throughout the night provide an easy and reliable way to collect temperature data; (2) Mobile phone technology allow to interpret the temperature curve using statistical models or machine learning techniques to identify the day of ovulation; (3) Other physiological parameters, such as cardiovascular function, respiratory rate and perfusion, may contribute to identifying the day of ovulation.

Basal temperature usually drops just before ovulation due to increased estrogen levels, then rises significantly at the time of ovulation due to increased progesterone levels caused by follicle luteinization taking place around the day of ovulation [10,11]. This increase is followed by a stabilization phase that lasts for about two weeks, or longer if pregnancy occurs. Despite the diversity of basal temperature curves around ovulation, statistical analysis often enables the identification of the day of ovulation to within one or two days [5].

The accuracy of temperature measurements on the upper limb may depend on the position of the sensor (proximal or distal; arm, wrist, or finger), the quality of the sensor itself, the movement data used to determine rest phases, and the algorithm used to identify temperature from a set of measurements taken during the night. Furthermore, identifying the day of ovulation using the temperature curve is only reliable if the correct statistical model is used [12,13,14].

Several articles report high accuracy in identifying the day of ovulation when other physiological parameters are combined with temperature [13]. Others show a similar level of accuracy in determining the day of ovulation using only temperature [6].

Tempdrop is a wearable device that monitors skin temperature via an armband sensor during sleep. It continuously measures axillary temperature using two integrated sensors (skin and microenvironment), along with a 3-axis accelerometer to monitor sleep–wake cycles. The Tempdrop app extracts and determines the basal body temperature (BBT) from the entire recorded sleep session. Then, the Tempdrop app interprets the temperature curve using a statistical model to identify the day of ovulation.

Another benefit of accurately measuring the basal body temperature each day with Tempdrop is that it provides information about the luteal phase. The temperature is known to reflects progesterone levels, which are frequently low in cases of infertility and in other contexts, such as premenstrual syndrome.

The aim of this study was to evaluate the accuracy with which this axillary temperature armband sensor identifies the day of ovulation and the fertile window, using the Clearblue Connected Ovulation Test System (Clearblue™, Swiss Precision Diagnostics GmbH, Geneva, Switzerland) as the reference method [15,16]. Another objective was to illustrate the variety of post-ovulatory basal body temperature patterns recorded by Tempdrop.

## 2. Materials and Methods

### 2.1. Ethics

The study received Institutional Review Board (IRB) approval on 27 March 2023, Informed consent was obtained from all participants, as documented in the consent form approved on 14 March 2023. Participants were informed about the study’s purpose, rights, and potential risks. Participation was entirely voluntary, with the option to withdraw without penalty.

### 2.2. Study Design

The research involved using the Tempdrop sensor and the Clearblue Connected Ovulation Test System. Participants used the app to record key data, including the first day of menstruation, results from the Clearblue Ovulation Test, and symptoms such as changes in cervical mucus, premenstrual symptoms, and cramps.

### 2.3. Inclusion Criteria

A total of 258 women aged 18–45 and residing in the United States were recruited to participate in the study, between April 2023 and June 2024.

Menstrual cycle data were included in the study only if at least 80% of days during the cycle had recorded temperatures, the cycle length was between 24 and 35 days, and the estimated post-ovulatory phase length was between 9 and 20 days. Of the 258 women recruited, 125 met the inclusion criteria, 69 women provided the data of two eligible cycles (at least 80% daily temperature) and 56 women provided one menstrual cycle data for analysis.

### 2.4. Estimated Day of Ovulation

The estimated day of ovulation was provided by both the reference (LH) and the armband sensor (Tempdrop).

#### 2.4.1. Reference Estimated Day of Ovulation

Only one positive Clearblue ovulation test per cycle was considered valid, except in cases where two consecutive positive tests were recorded, in which case the second positive test was designated as the actual LH surge. The reference estimated day of ovulation (LH-EDO) was defined as the day after the LH surge day.

#### 2.4.2. Armband Sensor Estimated Day of Ovulation

- Training phase

The algorithm was developed using a training dataset comprising a real-world sample of Tempdrop users who concurrently used ovulation predictive kits, including but not limited to the Clearblue system.

Basal body temperature (BBT) data were structured such that each menstrual cycle was represented by a sequence of 35 consecutive daily temperature values. In cases where cycles were shorter than 35 days, the final recorded temperature was repeated (padded) to ensure uniform input length across all samples —a prerequisite for processing with the machine learning model.

The dataset was randomly partitioned into training and validation subsets. Each cycle sequence was treated as a one-dimensional time-series signal, allowing the model to capture temporal dependencies and fluctuations in BBT associated with different phases of the menstrual cycle. This preprocessing enabled the application of a one-dimensional convolutional neural network (1D CNN) to extract relevant features and predict ovulatory patterns with improved accuracy.

The optimal model was retained as the stable algorithm and incorporated into the app.

- Testing phase

This article presents the results of the algorithm’s testing phase, based on the study described above. Tempdrop provides an estimated ovulation date (A-EDO). Importantly, the cycles used for testing were entirely independent and were not included in the training or validation datasets. We present the results of a retrospective algorithm, i.e., one that is applied after the end of a cycle. It was not possible to apply the algorithm prospectively in this analysis, i.e., to predict the day of ovulation before or immediately after it occurs, because not enough women contributed data for more than one cycle.

### 2.5. Peri-Ovulatory Period and Fertility Window

#### 2.5.1. Peri-Ovulatory Period

The peri-ovulatory period was defined as the A-EDO ±2 days. We apply this rule, following a previously published recommendation [5].

#### 2.5.2. Fertility Window

**Figure 1:**
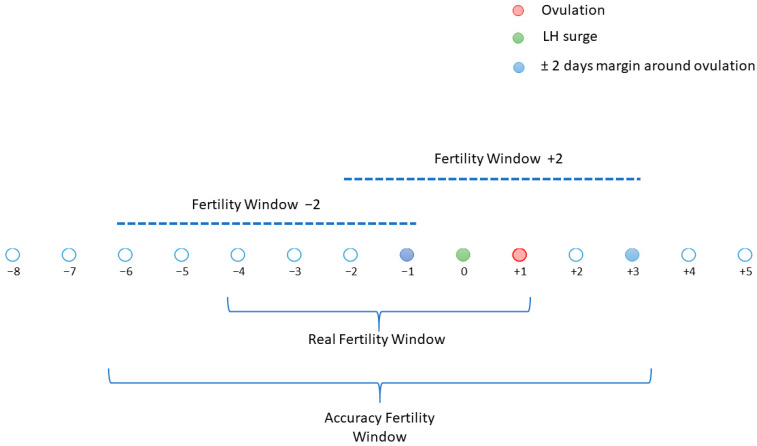
Presentation of the fertile window and accuracy of the fertility window.

The definition of the fertility window was based on its definition by Wilcox [15], which is the day of ovulation and the five preceding days, thanks to the presence of cervical mucus that allows spermatozoa to survive until ovulation. The accuracy fertility window was defined as the A-EDO ±2 days and five previous days, as shown in Figure 1.

### 2.6. Statistical Analysis

#### 2.6.1. Comparison of A-EDO with the LH-EDO

The distance between the A-EDO and the LH-surge day was tabulated and presented using a histogram. The average number of days between them was estimated as well as the standard deviation (s.d.). Then, the proportion of A-EDO falling within the LH-EDO ±2 days interval was calculated. Three figures were made to present (1) a random example of cycles where A-EDO falls within the LH-EDO ±2 days interval; (2) all cycles with delayed A-EDO and (3) all cycles in which A-EDO occurred before the detected LH surge.

#### 2.6.2. Performance Metrics for Detecting Fertile Days

- Some definitions:

True Positive (TP): Days correctly identified by the model as fertile, falling within the reference fertile window.

True Negative (TN): Days correctly identified by the model as non-fertile, lying outside the reference fertile window.

False Positive (FP): The model predicted days as fertile but falling outside the reference fertile window.

False Negative (FN): Days within the reference fertile window missed by the Tempdrop.

- Performance metrics

The ability of Tempdrop to identify fertile days was evaluated based on the following criteria:

Sensitivity (Recall) (1): The model can correctly identify fertile days among all reference fertile days (from LH-surge −6, included, to LH-surge +3, included), i.e., the proportion of correctly predicted fertile days out of all actual fertile days:

(1) $$\frac{TP}{TP+FN}$$

Specificity (2): The model can correctly identify non-fertile days (i.e., the proportion of correctly predicted non-fertile days out of all actual non-fertile days (i.e., outside the LH-surge −6 to LH-surge +3 interval)):

(2) $$\frac{TN}{TN+FP}$$

Accuracy (3): The proportion of correctly classified fertile and non-fertile days (True Positives (TP) and True Negatives (TN)) relative to all assessed days:

(3) $$\frac{TP+TN}{TP+TN+FP+FN}$$

Positive predictive value (4): A day identified by the sensor as being fertile is within the LH-surge −6 to LH-surge +3 interval

(4) $$\frac{TP}{TP+FP}$$

Negative predictive value (5): A day identified by the sensor as being non-fertile is outside the LH-surge −6 to LH-surge +3 interval

(5) $$\frac{TN}{TN+FN}$$

All analyses were performed with the R software (R version 4.4.1 The R Foundation for Statistical Computing).

## 3. Results

### 3.1. Comparison of the A-EDO and the LH-EDO

**Figure 2:**
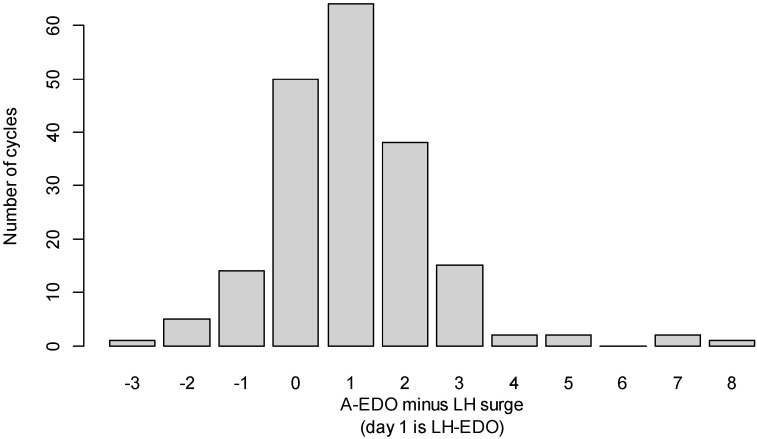
Distribution of differences between the A-EDO and the LH-surge.

Figure 2 presents the distribution of differences between the A-EDO and the LH-surge. The A-EDO occurred on average 1.02 (s.d. 1.7) days after the LH-surge, i.e., estimated LH-EDO without systematic bias.

In 181 out of 194 menstrual cycles (93.3%, 95% CI: 89–96%) the A-EDO was in the LH-EDO ±2 days interval, i.e., between LH-surge minus one and LH-surge plus three.

**Figure 3:**
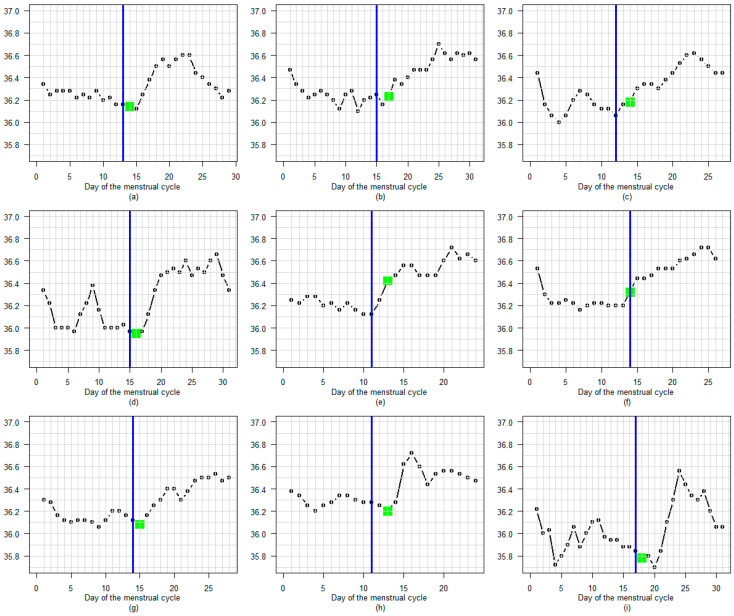
(**a**–**i**) Nine examples of cycles with a high degree of concordance between A-EDO and LH-EDO, selected at random from a total of 181. A blue line marks the LH surge day. A green dot indicates the A-EDO.

Figure 3 shows nine examples of cycles with a high degree of concordance between A-EDO and LH-EDO, selected at random from a total of 181. In these cycles, the detected LH surge occurs immediately before the increase in temperature.

**Figure 4:**
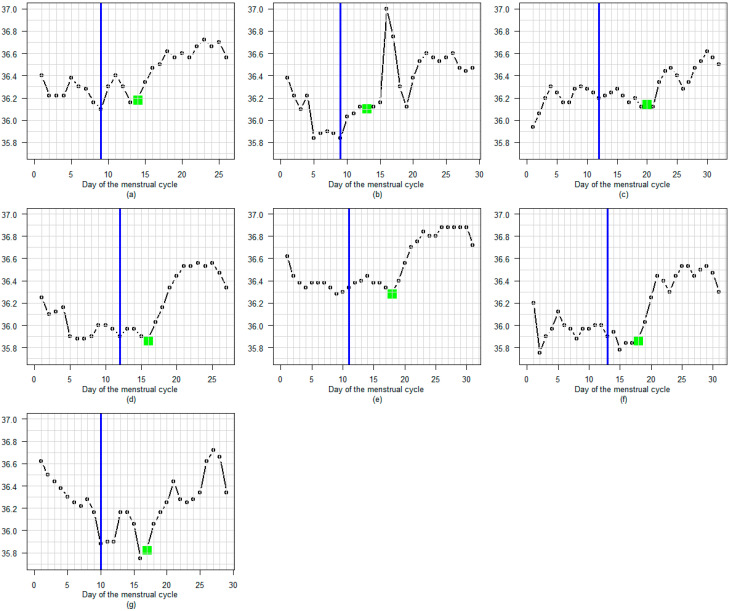
(**a**–**g**) The seven menstrual cycles with delayed A-EDO. In these cases, the LH surge appears to occur several days before the temperature rise. A blue line marks the LH surge day. A green dot indicates the A-EDO.

Figure 4 shows the seven menstrual cycles with delayed A-EDO. In these cases, the LH surge appears to occur several days before the temperature rise.

**Figure 5:**
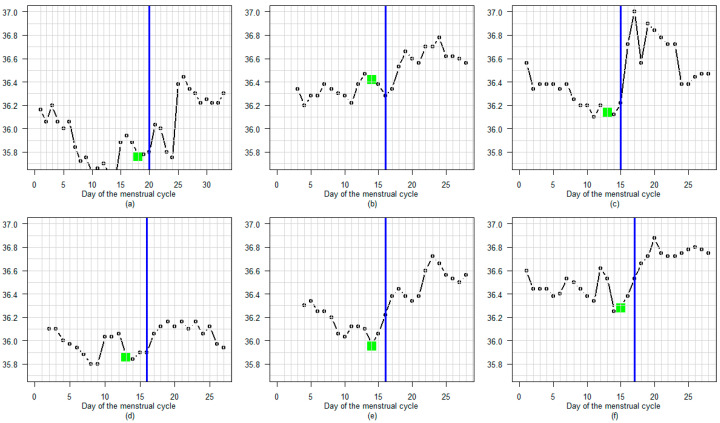
(**a**–**f**) The six menstrual cycles in which A-EDO occurred before the detected LH surge. A blue line marks the LH surge day. A green dot indicates the A-EDO.

Figure 5 shows the six menstrual cycles in which A-EDO occurred before the detected LH surge. In these cycles, temperature rises begin before the LH surge is detected.

### 3.2. Results of Performance Metrics for Detecting Fertile Days

Table 1 summarizes the performance metrics of Tempdrop in identifying fertile and non-fertile days.

Only 38 false positive days were observed, i.e., identified as fertile despite not being so. And, 38 false negative days were observed, i.e., not identified as fertile despite being fertile. The similarity between these two figures, which is due to sampling chance, produces an unusual result: sensitivity and positive predictive value are equivalent, as are specificity and negative predictive value. This should be noted but has no bearing on interpretation.

All performance indicators for detecting fertile days are high, especially specificity and negative predictive value.

### 3.3. BBT Profile During the Luteal Phase

**Figure 6:**
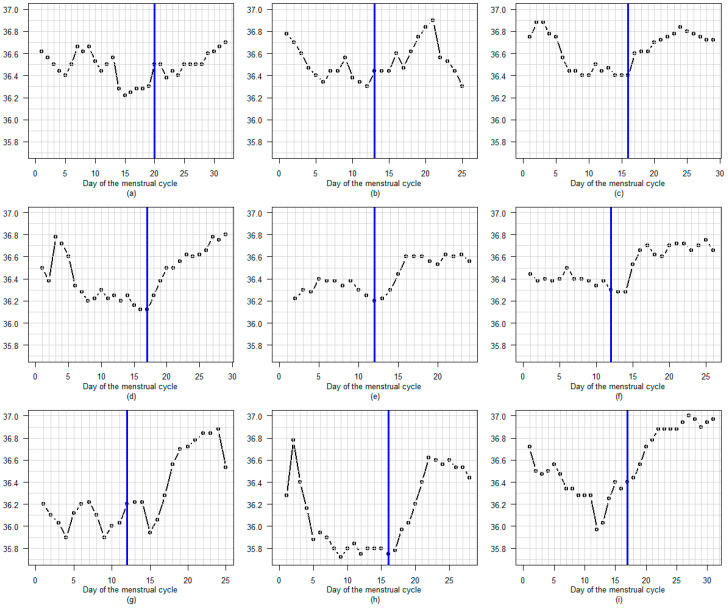
(**a**–**i**) Nine menstrual cycles show various levels of temperature rise from a low level before the LH surge to a higher level thereafter. A blue line marks the LH surge day.

**Figure 7:**
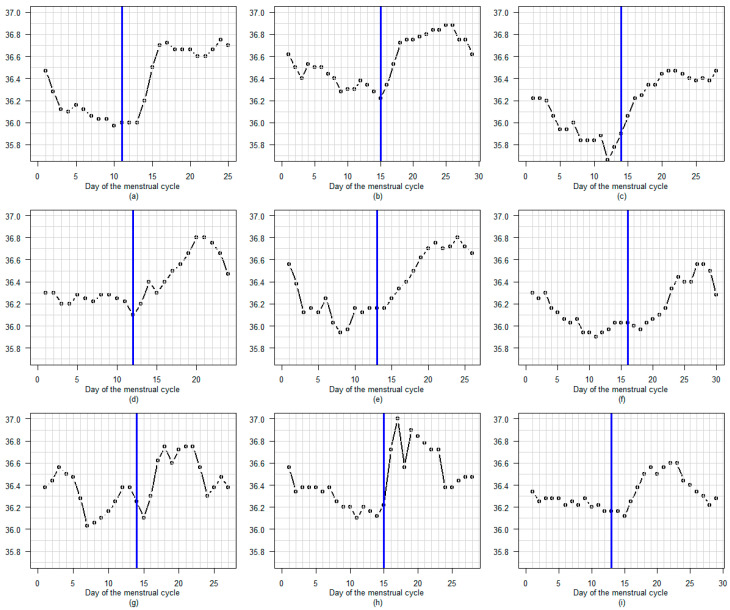
(**a**–**i**) Nine menstrual cycles show various shapes of temperature plateau during the luteal phase, with a blue line marking the LH surge day.

**Figure 8:**
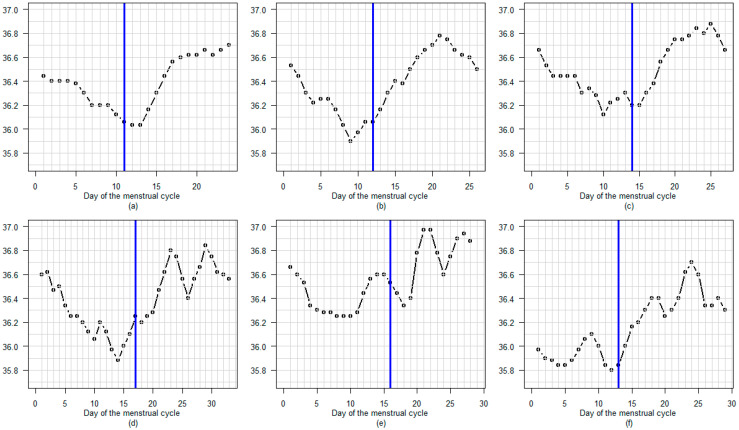
(**a**–**f**) The six menstrual cycles show various shapes of temperature plateau during the luteal phase. The blue line marks the day of the LH surge. Some temperature plateaus are regular, as shown above, while others fluctuate, as shown below.

Figure 6, Figure 7 and Figure 8 present some examples of BBT profiles with luteal phase temperature of diverse amplitudes and diverse shapes.

#### 3.3.1. Amplitude

Figure 6 illustrates the variation in the amplitude of the rise in BBT. The top three graphs depict menstrual cycles featuring a slight temperature increase around ovulation and a low plateau (approximately 0.1 to 0.2 °C). The middle three graphs depict cycles with slightly larger temperature increases (approximately 0.3 °C). The bottom three graphs show cycles with significant temperature increases (approximately 0.5 °C).

#### 3.3.2. Profile

Figure 7 shows how the shape of the BBT plateau varies during the luteal phase. The top three graphs show rapid and clear BBT rises at the beginning of the luteal phase. The middle three graphs show slow and delayed BBT rises during the first week after LH surge day. The bottom three graphs show cycles with a premature decrease in temperature, occurring several days before the end of the cycle.

#### 3.3.3. Regular or Fluctuating

Figure 8 shows how the BBT plateau evolved, day after day, during the luteal phase, being either regular (above) or fluctuating (below).

## 4. Discussion

This study was conducted to determine whether an armband device measuring the axillary temperature, specifically the Tempdrop, is accurate enough to be included in the list of alternatives to urine ovulation tests for determining the timing of ovulation. The results demonstrate high accuracy, and analysis of the differences between the LH test results and those obtained using this device suggests that these methods could be complementary. One of the results of this study is the clarity of the BBT profile as measured by the Tempdrop device around the time of ovulation and during the luteal phase.

Other devices, such as wristbands and rings, have already been shown to be a practical alternative to urine-based ovulation tests for determining ovulation times. Using a comparable methodology, the performance metrics observed in our data are close to those presented in Niggli et al.’s paper for a wristband device (see their Table 3) [5]. They estimated accuracy to be 93%. We estimated accuracy to be 98.6% for Tempdrop. In their recent paper comparing ovulation days detected using temperature curves obtained from a ring with those detected using the calendar method, Thigpen et al. estimated that the ring device’s estimation was correct in 96.4% of cycles [6].

Basal body temperature (BBT) is defined as the lowest body temperature a person naturally reaches when at rest, typically, during a 24 h circadian cycle. This has been shown to reflect hormonal activity. During the menstrual cycle, BBT increases after ovulation, which can be used to identify the day on which it occurs. Traditionally, BBT is measured in the mouth, rectum or vagina right upon waking, before getting out of bed, after at least 3 h of sleep, ideally after the night’s sleep, not an afternoon nap.

New technologies offer an alternative solution by tracking nightly temperature variations and measuring the temperature externally on the skin during night-time sleep. Two conditions must be met: rest phases during sleep must be identified, and a place on the skin must be found where the temperature will not be affected too much from the environment. In addition, algorithms are used to correct the measurement when environmental conditions are affecting the measurement site. Like other fertility trackers that measure wrist temperature (WST) during sleep or rings measuring the temperature changes on the person’s finger, Tempdrop measures the axillary (armpit) temperature, and uses an acceleration sensor and a dedicated algorithm to identify rest phases.

The Tempdrop device features two major innovations. First, it measures the temperature of the skin above the axillary artery. This location is one of the places where skin temperature most closely resembles internal body temperature, as it is near a large blood vessel and if the measurement is taken for more than 12 min, it closely corresponds with the core body temperature. In addition, the device has a second temperature sensor that measures the ambient temperature. This enables the device to identify periods when the arm is closed and in contact with the chest, i.e., when the temperature measurement is most accurate.

Our results show that the Tempdrop performs remarkably better than the BodyMedia SenseWear armband sensor, which was tested ten years ago [7]. The authors observed a relatively poor correlation between BodyMedia SenseWear and oral thermometer temperature readings. Unlike Tempdrop, the BodyMedia device is worn on the outside of the upper arm and not in the armpit or near a large vessel. The Tempdrop sensor measures the temperature of the skin above the axillary artery and also contains a microenvironment temperature sensor in its tip that is not in direct contact with the skin. This sensor filters out ‘temperature noise’ and serves as a reference for the area temperature. Additionally, during sleep, the sensor is mostly thermally isolated because the arm is closed for most of the time, which significantly reduces environmental effects on the measured temperature and allows for higher temperature accuracy. The Tempdrop device is an axillary temperature sensor with specific characteristics that are likely essential for accurate temperature measurement. The fundamental objective of new technologies in this context is to make daily temperature measurement more practical for determining ovulation timing and obtaining a clear temperature curve that can be used to evaluate luteal phase quality. Several sensors have been proven to be accurate in determining ovulation timing. These sensors are placed in different anatomical locations, such as the vagina or upper limb. The Tempdrop axillary temperature wearable sensor has two features that potentially allow it to outperform other devices: first, its position close to the axillary artery, which communicates the central body temperature to the skin; and second, the addition of a second temperature sensor that identifies periods when the arm is on the chest. This provides a second check of the minimal impact of environmental temperature. Nevertheless, only further studies could confirm that the axillary temperature wearable sensor outperforms others.

Previously, the BBT method was not considered accurate enough to determine the day of ovulation [17,18,19,20,21,22,23]. It was mainly used to confirm that ovulation had occurred, rather than to identify the exact day on which it occurred [24,25]. The day on which the temperature was lowest, just before the BBT rise (i.e., the nadir), was generally considered to be the day closest to ovulation [26,27]. This low temperature reflects the high estrogen level just before ovulation. The subsequent rise in BBT confirms ovulation by reflecting the rapid increase in progesterone [11]. However, a clear nadir is not present in all cycles [27]. The lowest temperature may occur several days before ovulation. In some cycles, the temperature rise begins one or a few days before ovulation, with progesterone secretion beginning beforehand. Finally, in some cases, the temperature rise is delayed by one or a few days after ovulation due to progesterone levels rising slowly [28]. These cycle peculiarities explain why the nadir is not always a perfect method of locating ovulation. Recently, BBT has gained renewed interest due to the possibility of using a statistical model to identify the day of ovulation, which is made possible by mobile phone technology. Such an algorithm can adapt to the variety of BBT curves. This is probably one of the reasons for the high accuracy observed when using appropriate devices with optimized algorithms, such as those used by Tempdrop.

A recent paper describes how machine learning statistical models can be used to identify the phases of the menstrual cycle when several physiological parameters, such as temperature, cardiovascular function, respiratory rate, and skin perfusion, are considered together [14]. We can see from the comparison that the axillary temperature armband sensor we tested performs comparably or better. This may be due to the direct link between progesterone and temperature [11], but to a more complex, indirect process that explains changes in cardiovascular function, respiratory rate, and skin perfusion during the cycle (see their Figure 1) [9].

One unexpected result of our study was the distinct high-temperature profile observed during the luteal phase, as illustrated in the figures. Without comparing these results with those obtained using other devices (such as ear, vaginal, wristband or ring devices), it is impossible to determine whether the tested armband device is more effective or equivalent. Nevertheless, this is sufficient to prompt research into the potential benefits of using the BBT profile during the luteal phase to identify luteal deficiency for use in clinical practice [29,30,31,32,33]. In a study comparing levels of the progesterone metabolite PDG and basal body temperature (BBT) throughout the female cycle, we observed a strong correlation between the two [11]. However, once the PDG level exceeds a certain threshold, temperature no longer rises. Therefore, BBT only provides information about progesterone levels when they are low. A delayed increase in BBT may indicate a delayed rise in progesterone after ovulation (see Figure 7). A decrease in BBT several days before the onset of menstruation may suggest a premature drop in progesterone levels. A low BBT plateau may also indicate low progesterone levels (see Figure 6). An irregular BBT plateau may suggest unstable progesterone levels during the luteal phase see Figure 8). The quality of the BBT curve obtained using the Tempdrop device could facilitate monitoring of the luteal phase in clinical practice. This would be particularly useful for women with premenstrual syndrome or polycystic ovarian disease, and for subfertile couples [34,35,36]. Note that a blood sample would still be useful seven days after the LH or mucus peak day. High levels of progesterone at this stage are considered beneficial for increasing the chance of conception [37].

This study focused on performance evaluation. It did not evaluate the practicality of the device. This is a limitation. A further study might evaluate its feasibility, notably in different populations. The main limitation of our study is the lack of evaluation of the performance of the algorithm used prospectively, i.e., on a daily basis before the high plateau was established. However, experience gained by the authors testing other comparable devices shows that the performance obtained prospectively is similar [5]. A further study is planned to verify whether the same applies to Tempdrop. The concordance of days of ovulation assessed by the armband device and by the LH-surge was high but not perfect. Due to the clarity of the temperature rise measured by the axillary temperature wearable device, the rare cases of discrepancy might be physiological: a high LH is sometimes observed several days before ovulation; LH can also reach the threshold after ovulation, when it is maintained at a high level to promote better luteinization.

## 5. Conclusions

In conclusion, our results demonstrate that this axillary temperature armband sensor is an effective alternative to urine ovulation tests for determining the timing of ovulation. Another advantage is that it provides a clear temperature curve that can be used to evaluate the quality of the luteal phase.

## Figures and Tables

**Table 1:** Performance metrics of fertility days (5394 days of menstrual 194 cycles).

Performance Metrics	Numbers	Percentage (95% Confidence Interval)
True positive days	1138	
True negative days	4180	
False positive days	38	
False negative days	38	
Sensitivity	1138/(1138 + 38)	96.8% (95.6; 97.7)
Specificity	4180/(4180 + 38)	99.1% (98.8; 99.4)
Accuracy	(1138 + 4180)/5394	98.6% (98.2; 98.9)
Positive predictive value	1138/(1138 + 38)	96.8% (95.6; 97.7)
Negative predictive value	4180/(4180 + 38)	99.1% (98.8; 99.4)

## Data Availability

Up to 36 months after article publication, individual participant data underlying the results, after deidentification, could be provided to researchers with a methodologically sound proposal approved by an independent review committee, to achieve their aims.

## Abbreviations

The following abbreviations are used in this manuscript:

BBT	Basal Body Temperature
IRB	Institutional Review Board
LH	Luteinizing Hormone
A-EDO	Armband-Estimated Day of Ovulation
1D CNN	One-Dimension Convolutional Neural Network
s.d.	Standard deviation
TP	True Positive
TN	True Negative
FP	False Positive
FN	False Negative
